# Intricacy, Symmetry, Diversity

**DOI:** 10.3201/eid2607.AC2607

**Published:** 2020-07

**Authors:** Byron Breedlove

**Affiliations:** Centers for Disease Control and Prevention, Atlanta, Georgia, USA

**Keywords:** art science connection, emerging infectious diseases, art and medicine, about the cover, Pierre Joseph Redouté, Iris Germanica, les liliacées, intricacy, symmetry, diversity, mosaic virus, emerging viruses, viruses, flowers, irises, Biodiversity Heritage Library

**Figure:**
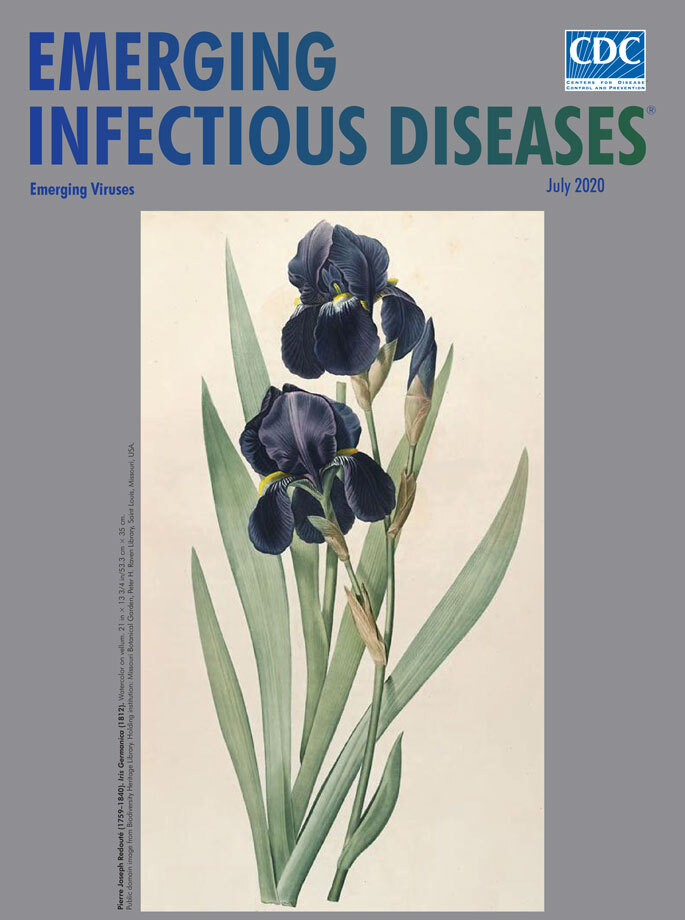
**Pierre Joseph Redouté (1759–1840). *Iris Germanica* (1812)**. Watercolor on vellum. 21 in × 13 3/4 in/53.3 cm × 35 cm. Public domain image from Biodiversity Heritage Library. Holding institution: Missouri Botanical Garden, Peter H. Raven Library, St. Louis, Missouri, USA.

Belgian artist Pierre-Joseph Redouté is the “most celebrated flower painter of quite possibly the entire history of botanical art” and “remembered as one of the greatest botanical illustrators in history,” notes Grace Costantino, from the Biodiversity Heritage Library. Redouté, whose patrons included members of French royalty and other luminaries interested in botany, published more than 2,100 plates depicting more than 1,800 species of flowers, many of which had never been illustrated before. 

Born in 1759 at St. Hubert in the Belgian province that is now Luxembourg, Redouté was descended from a family of Belgian painters. He trained in his father’s studio, and by the time he was 13 years old, Redouté was earning his living as an artist. In 1782, he teamed with his elder brother, Antoine-Ferdinand, to design the stage scenery for the Théâtre-Italien in the rue de Louvois, Paris. There Redouté started painting flowers during his free time and studied with Dutch artist Gerard van Spaendonck, a professor of flower painting at the Muséum National d’Histoire Naturelle. Spaendonck taught the young Redouté engraving and watercolor techniques, which he continued to use and refine. Artist and author Wilfrid Blunt asserts that Redouté owes much of his success to those discoveries and describes Redouté’s technique as using “pure water colour, gradated with infinite subtlety and very occasionally touched with body-colour to suggest sheen.” 

The image *Iris germanica* featured on this month’s cover comes from *Les liliacées*, a misleading title for a volume of prints that also features many other plants. This collection was published while the artist was under patronage from the Empress Joséphine, first wife of Napoleon, and is considered to be Redouté’s masterpiece. 

The plant’s gentle intricacy and distinctive three-fold symmetry are apparent in the two indigo blooms, each shown from a different perspective, paired with a third stalk poised to bloom. Faintly striated sword-shaped leaves fan out around the stalks and petals, tapering into finely pointed tips. The upright petals, or standards, and the pendant petals, or falls, are the same color. Author and gardener Steve Bender notes that “Standards and falls may be the same color or radically different colors. Dream up any color combination, and you can probably find it.”

Named for the Greek goddess Iris, who traveled along rainbows to deliver messages between the gods and the Underworld, the genus *Iris* comprises more than 300 species and perhaps 50,000 varieties of flowers. Flowering irises fill gardens with an assortment of eye-catching colors in the spring and summer and have been the subject of many well-known paintings by renowned artists.

The Missouri Botanical Garden notes that “the presumed father of most modern bearded iris cultivars,” *Iris germanica*—which is not actually native to Germany but rather to the Mediterranean and central Asia—has become established throughout the world and naturalized throughout much of Europe and the United States. Irises are diverse in their ecology, flourish under myriad conditions, and have increased in variety and geographic range largely because of human activity. 

Although generally hardy, irises are susceptible to a number of viruses, including Iris mild mosaic virus, iris severe mosaic virus, iris fulva mosaic virus, bean yellow mosaic virus, narcissus latent virus, and the more recently documented cucumber mosaic virus, broad bean wilt virus, and tobacco ringspot virus. Irises can be affected by multiple viruses at the same time, and the viruses are typically spread by aphids. 

During the late 19th century, about 50 years after Redouté died, Adolf Eduard Mayer showed that tobacco mosaic disease could be transferred among plants. Dmitry Iosifovich Ivanovsky discovered a microscopic infectious agent that could permeate porcelain Chamberland filters (bacteria could not). And Martinus Beijerinck replicated Ivanovsky’s discovery and called this new pathogen “*contagium vivum fluidum.*”

Since that time, rapid transit, global trade, urbanization and destruction of natural habitats, and modern agricultural practices have accelerated the emergence and spread of viruses among humans, animals, and plants. As the landmark 1992 Institute of Medicine report on Emerging Infections reminds us “. . . in the context of infectious diseases, there is nowhere in the world from which we are remote and no one from whom we are disconnected.” The emergence of SARS-CoV-2 underscores how connected the modern world is, and shows that, like the artist’s iris, emerging viruses continue to expand their diversity and range. Spending a few quiet moments enjoying Redouté’s striking botanical illustration may offer a brief respite to public health professionals, clinicians, and researchers engaged in detecting, preventing, and responding to emerging viruses.
